# Educational behaviors of pregnant women in the Bronx during Zika’s International emerging epidemic: “First mom … and then I’d Google. And then my doctor”

**DOI:** 10.1186/s12884-021-04170-0

**Published:** 2021-10-26

**Authors:** Miguel Rodriguez, Antoinette A. Danvers, Carolina Sanabia, Siobhan M. Dolan

**Affiliations:** 1 Department of Family Medicine, Loma Linda University Health Educational Consortium, Loma Linda, CA USA; 2 Department of Obstetrics, Gynecology and Women’s Health, Albert Einstein College of Medicine / Montefiore Medical Center, Bronx, NY USA; 3 Department of Obstetrics, Gynecology, and Women’s Health, Montefiore Medical Center, 1695 Eastchester Road, Bronx, NY 10461 USA; 4 Department of Obstetrics, Gynecology and Reproductive Science, Icahn School of Medicine at Mount Sinai and Mount Sinai Health System, New York, NY USA

**Keywords:** Zika virus infection, Health education

## Abstract

**Background:**

The objective of the study was to understand how pregnant women learned about Zika infection and to identify what sources of information were likely to influence them during their pregnancy.

**Methods:**

We conducted 13 semi-structed interviews in English and Spanish with women receiving prenatal care who were tested for Zika virus infection. We analyzed the qualitative data using descriptive approach.

**Results:**

Pregnant women in the Bronx learned about Zika from family, television, the internet and their doctor. Informational sources played different roles. Television, specifically Spanish language networks, was often the initial source of information. Women searched the internet for additional information about Zika. Later, they engaged in further discussions with their healthcare providers.

**Conclusions:**

Television played an important role in providing awareness about Zika to pregnant women in the Bronx, but that information was incomplete. The internet and healthcare providers were sources of more complete information and are likely the most influential. Efforts to educate pregnant women about emerging infectious diseases will benefit from using a variety of approaches including television messages that promote public awareness followed up by reliable information via the internet and healthcare providers.

**Supplementary Information:**

The online version contains supplementary material available at 10.1186/s12884-021-04170-0.

## Background

Emerging Zoonotic infectious diseases, such as Coronavirus in 2019-2020, are expected to increase among human populations, posing public health challenges. International travel, along with climate change and human population growth, are fueling changes in global ecology leading to this increased transmission [1–3]. In 2016-2017, Zika was one emerging infection with risk for poor outcomes in pregnant women due to increased risk for teratogenic effects. Because regions with high rates of circulating virus in 2016-2017 included the Caribbean and Latin America, pregnant women in the Bronx were at high risk of travel associated Zika infection [4]. New York State had the second highest number of cases with a reported incidence of 1133 cases (1/1/2016 - 12/31/2017). The Bronx, a diverse borough with high rates of poverty in New York City, was the most affected with 394 reported Zika cases [5]. As a new infection in the Americas, Zika created a need for rapid dissemination of information about the risks of Zika transmission and prevention strategies, especially for pregnant women.

Educational efforts included a 3 year, $21 million plan by the New York City Department of Health (NYCDOH) to protect against Zika starting in 2016 which incorporated a citywide Zika Awareness Campaign on the risks of transmission via travel and sex. The campaign included messaging on multiple platforms including TV, social media, and newspapers. Furthermore, in 2016, the NYCDOH conducted more than 220 community presentations, distributed over 4,000 Zika awareness kits, and issued over 2,000 Zika travel warning posters [6]. Based on models identifying areas for greatest risk of Zika importation, public education was targeted in the Bronx and upper Manhattan, which is also home to many immigrants from the Caribbean as well as South and Central America [7]. The objective of this qualitative research in our urban medical center to learn how pregnant women seeking prenatal care learned about Zika and the sources of information that influenced their understanding in order to guide recommendations for future messaging around emerging infections.

## Methods

The participants in the study were recruited at prenatal care visits in the Bronx, New York which is a diverse inner-city community with a largely immigrant population. Prenatal care is delivered by OB/GYN physicians and physician assistants. During the Zika epidemic, patients presenting for prenatal care visits were routinely asked about travel to Zika endemic countries. Those who were identified to be at risk for Zika exposure based on recent travel were offered counseling regarding risk of Zika infection and potential effects on pregnancy and diagnostic testing for Zika infection per CDC guidelines [8]. Study participants were selected from the group of women with possible Zika exposure who underwent diagnostic testing in the Summer of 2017.

Women eligible to participate in the study were pregnant, 18 years or older, and spoke English or Spanish as their preferred language. All participants provided written informed consent, completed a questionnaire providing demographic information and then participated in a recorded face-to-face interview. Each participant was given a $40 gift card as compensation for her time.

Participants were interviewed for approximately 20-30 minutes by a member of the research team who conducted a semi-structured in-person interview in the patient’s preferred language. Nine interviews were conducted in English and 4 in Spanish. The interviews were audio-recorded and transcribed verbatim. Interviews conducted in Spanish were translated into English and transcribed. We used an interview guide to gather information about participants’ knowledge and understanding of Zika infection, their experience with Zika education, prevention, counseling, and testing as well as their attitudes toward recommendations related to Zika infection. Finally, we asked the participants to share insights about how to improve communication so that accurate information about Zika could reach the wider community.

We analyzed the transcripts using a grounded-theory approach [9]. We began by entering all transcripts into Dedoose version 4.5 (2017), which is “a web application for managing, analyzing, and presenting qualitative and mixed method research data” [10]. The data were coded individually in Dedoose by three members of the research team. Upon initial review of transcripts for repeating ideas, words and frequently used phrases, a set of codes was developed in a preliminary codebook. The preliminary codebook was further developed by analyzing the full set of coded transcripts in Dedoose. The full set of codes was organized into major themes and thematic saturation was reached after completing 13 interviews. *Understanding Zika Virus as an STI* was previously published based on three themes amongst the findings of this qualitative study [11].

## Results

The demographic data describing participants is presented in Table 1. The median age of the participants was 29 years, 12 participants already had at least one child and 11 were residents of the Bronx. We had a mixed racial composition and all but one of the participants identified as Latinx/Hispanic.

**Table 1:** Baseline characteristics of the participants in the study

	Participant demographic characteristics (***N***=13)				
Variable	N	%	Variable	Median	Range (min, max)
**Gender**			**# of Pregnancies**	**3**	**(1,6)**
Female	**13**	**100%**	**# of Kids**	**1**	**(0,2)**
**Ethnicity**			**Age**	**29**	**(18,39)**
Latino / Hispanic	**12**	**92%**			
Non -Latino / Non -Hispanic	**1**	**8%**			
**Self-reported race**					
Black / African American & White	**4**	**30%**			
White	**1**	**8%**			
American Indian / Alaskan Native	**1**	**8%**			
Black / African American & American Indian / Alaskan Native	**1**	**8%**			
Native Hawaiian / Pacific Islander	**1**	**8%**			
Other / Did not respond	**5**	**38%**			
**Language**					
Spanish	**4**	**30%**			
English	**9**	**70%**			
**Civil status**					
Married	**7**	**54%**			
Cohabiting	**4**	**30%**			
Single	**2**	**16%**			
**Education**					
GED	**1**	**8%**			
Some College	**7**	**54%**			
College Degree	**4**	**30%**			
Graduate Degree	**1**	**8%**			
**Estimated Annual Household Income**					
Less than $25,000	**4**	**30%**			
$26,000 - $50,000	**1**	**8%**			
$51,000 - $80,000	**2**	**16%**			
Greater than $80,000	**3**	**23%**			
Refused / other	**3**	**23%**			
**Employment status**					
Full Time	**6**	**47%**			
Part Time	**4**	**30%**			
Unemployed	**3**	**23%**			
**Type of insurance**					
Medicaid	**8**	**62%**			
Medicare	**1**	**8%**			
Commercial	**4**	**30%**			
**County of residence**					
Bronx	**11**	**84%**			
Manhattan	**1**	**8%**			
Rockland	**1**	**8%**			
**Zika tested**					
Yes	**13**	**100%**			
**Zika test results**					
Negative	**13**	**100%**			

### Themes

We asked participants how they learned about Zika during pregnancy. They discussed a variety of information sources and noted that each played a different role, ranging from providing awareness, providing knowledge, and providing support. Our analysis identified three main themes that are presented here: (1) TV was the main source of exposure to Zika information, (2) Google was the primary place to look up information, and (3) Healthcare providers were the main source of personalized guidance and validation (see Table 2).

**Table 2:** Characteristics of main informational sources used

	TV	Internet	Doctors / Clinics
**Advantages**	* Wide Reach - All Ages * Trusted * Creates Motivation / Concern	* Immediately Available * Lots of Information	* Personal Support * Trusted * Actionable
**Barriers**	* Limited Information * Time dependent	* Validity / Trust * Scary - No personal support	* Not immediately available
**Function**	Exposure	Research	Guidance

## Theme 1: TV was the main source of exposure to Zika information

The initial source of information about Zika was most often through television. Ten participants stated they learned about Zika by watching TV, and this included all 4 women who were interviewed in Spanish. Most mentioned watching news broadcasts, and many stated that they watched the news on Spanish language channels.

‘I heard it on television… There are Dominican channels’ (29 year old woman, traveled to Dominican Republic)

‘On television. I remember it came out on the news…I think it was Telemundo 47’ (18, traveled to Dominican Republic)

Sometimes participants did not learn about Zika by watching television themselves, but through a third party like a parent or grandparent who recounted the information they had seen and heard on TV. They talked about the wide reach of TV as a multi-generational source of information for their families and friends. This was a source of information that the community trusted.

‘My mother believes everything she hears on TV… whatever is on TV is what – it’s the truth…she’s older and watches TV everyday (28, husband traveled to Puerto Rico)

“When I was pregnant… I’ll watch the news of whatever clip it was on Zika…it was scary but everyone looked like – everyone was in the same page, you know? Everybody has the same general idea of what Zika is. (31, traveled to Honduras)

The repeated messaging from television affirmed the importance of Zika awareness for pregnant women, for whom it raised the greatest medical concerns. As a result, TV went beyond educating the community to also fostering interest and motivation in considering Zika prevention.

‘It’s always the same with any publicity campaign they do. People ignore it. They don’t think they need to waste their time looking at it. And they don’t trust it. They always think it’s all about money. But, later, when more news comes out on television, that’s when they want information.’ (18, traveled to Dominican Republic)

‘[I knew] all of this just from a flyer [seen at the clinic]. And then I started watching it on TV and I'm like, “I know about this. You see, I told, I told you about Zika.” [Laughter] And everybody was like calling me, “What’s that that’s coming? Oh my God, everybody is telling about this.” And, “Yes, it’s great.” ‘(31, traveled to Honduras)

With a short news cycle, repeated messaging early on in the epidemic caused a great awareness, but the lack of sustained messaging left some women wondering if Zika was still a concern. Some participants talked about the rise and fall of Zika information in the news and were unclear about the threat it posed during this study in the Summer of 2017 when TV messaging had waned.

‘Maybe it has gone away. I don’t know. Because there was more talk before. They used to talk about this on the news, but now they are not talking about it.’ (29, traveled to Dominican Republic)

‘They showed a lot of – when everything was starting, like that was maybe like last year or the year before that they show a lot of babies that were born with deformities, you know.’ (25, traveled to Ecuador)

The information from television was often too succinct. Participants felt they received some information but not the whole story and they reported that they did not feel knowledgeable about Zika.

‘And then they’re like this whole, big news story for months and then it goes away. And then you don’t hear about what, you know – how the – how this affects their child uhm, you know, how has it affected the parent, like the livelihood of the mother, the – the uhm, the livelihood of the – of the – of the child, whether or not the child will survive’ (30, traveled to Saint Thomas)

In addition, the dramatic presentations of affected babies that were initially shown on TV to engage viewers often scared pregnant women. Participants reported seeing scary images on TV and some women turned to denial to cope with the anxiety.

‘In the news they show the worst part of it – you know, only the birth’ (25, traveled to Ecuador)

‘Sometimes they show things on the news that you think won’t happen to you, that you’re somehow safe.’ (36, traveled to Dominican Republic)

### Theme 2: Google was the primary place to look up information

After learning about the existence of Zika from a source like television, either directly or through a family member, our participants often felt the need to learn more. Ten participants explained how they used Google to search the internet to learn more about Zika; it was their immediate first step to find out more information. For some it was one of the only sources of information available to them. Many women also used Google because it was easily accessible on their phones. Most importantly, they felt it was more informative than their social networks.

Interviewer: Where have you gone? … you said you looked it up.

Respondent: ‘Yeah. Uhm, Google is my best friend. So, I Google everything.’ (39, partner traveled to Mexico)

Interviewer: What are you gonna do on the internet?

Respondent: ‘Read, Google.’

Interviewer: Are you gonna ask anyone about it?

Respondent: ‘No because I’m pretty sure nobody around me is gonna know just like – just like I didn’t.’ (30, traveled to Puerto Rico)

‘I feel like the media and technology is in the palm of your hands. So, I think it’s like it’s so easy to just – just type it and you just research it. You could do some research of it.’ (39, partner traveled to Mexico)

‘On my phone [ I use] Google…Cause I’m always on my phone or on the go.’ (22, traveled to Mexico)

Accessibility to healthcare information on the internet gave participants immediate access to a broad range of detailed information. This allowed search engines like Google to become their primary source of information, well before they have spoken to a clinician or visited a healthcare setting. Participants shared they were likely to use Google to look up information both before and after consulting with their doctors.

‘First the mom [who, found out via TV] and then I’d Google. And then my doctor ...Any questions that I have that I wanna ask the doctor…I’ll just ask to Google and I’ll try to learn more that way.’ (39, partner traveled to Mexico)

‘Every time I go [to a doctor’s appointment], I know that if I want to learn more, I’ll have to look up the information [on Google]. That’s just how I am. That’s me.’ (36, traveled to Dominican Republic)

Participants also mentioned using the internet as a way to share information with others. Images of affected children were an important part of Zika education and used by some to educate their partners.

‘I had to go online and show him … I had to show him pictures…of what could possibly happen.’ (22, traveled to Mexico)

While some women relied heavily on the information they received online, other women expressed uncertainty about the quality of this information. In the presence of conflicting information, the internet felt like a source of potential misinformation to some participants.

‘I feel when people start Googling things or doing internet research, it’s not reliable.’ (28, partner traveled to Puerto Rico)

‘That’s a problem because you don’t know whether you can trust a site or not. Sometimes you read contradictory ideas.’ (36, traveled to Dominican Republic)

Some participants found the images and messaging on the internet to be too negative and felt it conveyed a sense of hopelessness. The images found on Google also created a sense of fear and anxiety for participants, especially about the health of their baby.

‘I went to Google… it was pretty scary because it becomes graphic. They show pictures of the baby’s birth defect.’ (25, traveled to Ecuador)

‘I did like research…I was in Google. So, it’s like all negative. They give you no positive. It’s as if you’re going to die… All those horrible things they showed me.’ (31, traveled to Honduras)

‘I just didn’t trust going on the internet and then it makes me more paranoid so I’d rather just stay away.’ (28, partner traveled to Puerto Rico)

## Theme 3: Healthcare providers were the main source of personalized guidance and validation

Healthcare providers guided women with personalized recommendations and information that women found to be highly reliable. For many immigrant women at risk, access to healthcare settings are not readily available until they become pregnant. For some participants, they did not access the healthcare setting until after becoming pregnant and the window of opportunity for prevention had passed because they had not received any guidance about how to avoid exposure prior to pregnancy (11).

“I know that the information is available. I want to get it from a reliable source which would be a doctor… [the doctor gave] me a paper on Zika so I read that. I was comforted by that pamphlet that they gave me.” (28, partner traveled to Puerto Rico)

Healthcare providers were noted to be important for providing additional validation regarding the seriousness of the infection and the possibility of Zika as a threat to each woman’s individual health. While the information raised concern for the participants, it did not always lead to participants implementing preventive measures and they remained at risk for Zika exposure.

‘After they [ the doctors] talked to me a bit about it [Zika], I started searching on Google, and that made me worry a little more… but there’s nobody in my family, I don’t know of anyone who has had a problem [with Zika], so, I wasn’t so terribly worried. But, now that I am pregnant, and my doctor talked to me, it seems more important to me.’ (36, traveled to Dominican Republic)

This affirmation was important given the fact that, for some participants, the extreme images they saw on TV and the internet seemed unlikely. But some felt their interaction with the doctor was more informative and valued than the information gleaned from TV, internet and social media.

‘[The doctor says] ‘here’s some information. This is Zika.” [But she should also say] “This is a picture of what would happen if you get infected by Zika.” …I think they should be more adamant about letting us know…I think they [doctors] feel like maybe they [pregnant women] already knew because it’s been out there so much like on social media and stuff with, you know, TV and stuff like that, the news. That I think they feel like we already know.’ (31, traveled to Honduras)

The healthcare setting was the most valued source of information for our participants because all of our participants had already risked exposure to Zika, given the timing of their travel or their partner’s travel.

## Discussion

Our qualitative analysis of interviews with pregnant women at risk of travel associated exposure to Zika uncovered a variety of information sources used. We discovered a general trend in how women learned information that followed a path of three themes: 1) TV as a main source of exposure to Zika information, 2) Google for internet-based research on Zika, and 3) individualized guidance and reinforcement in healthcare settings. Our participants’ understanding about Zika evolved as they became exposed to these different sources of information.

Television news outlets provided the initial awareness of Zika for most pregnant women and their families, but it was presented in broad brushstrokes and with limited detail. Several of our participants identified Spanish language channels as a trusted source of information, which removed the language barrier for themselves and their families. Individuals at home, who spent more time watching TV, recounted the information to family members and reached a large part of pregnant women’s social network. Television was impactful with its display of graphic images representing the effects of Zika on newborns. The buzz created by TV reached pregnant participants via family members, partners, and friends who watched the news and reached out to them. It provided a sense of urgency and motivation to act for the community. Presentation of Zika in news outlets made our participants feel fearful through graphic images without clarity around prevention measures that may provide hope. For some participants, the extreme images made them feel disconnected from and distrustful of the information they were receiving. Although the news included information on prevention and resources, its role was mostly as a first line exposure that generated interest and curiosity. However, the news was also a time-dependent informational source. The interest television generated around Zika waned after a few months. As a result, some women thought Zika was no longer relevant or urgent at the time they were interviewed for this study, even though travel related exposure continued to pose medical challenges to pregnancy.

Women went to the internet and used Google to learn more about Zika. After an initial exposure, most often from TV, women used Google to learn more and get more detailed information. The ease and immediate availability of information, made it their “best friend” as they could access tons of information in the palm of their hand. In cases where there was no one to ask, they could ask Google. They could also share information with others via the internet. However, this breadth of information could include contradictory messages and often left participants struggling to determine their validity. Some women only trusted government sites or sites with clinical information such as WEBMD. Searching online can be a lonely experience and without greater support, some women did not even look up Zika on Google as they were afraid of the scary images it would present.

Contact with the healthcare system was noted to be vital for clarity, affirmation and education. In our study, participants acknowledged that despite the breadth of information available through different media sources, they needed their prenatal care provider to confirm what they had learned. Clincians provided an additional level of trusted guidance and support. It is important to continue to attempt to break down barriers that immigrant women may face in accessing healthcare providers so that they can review and personalize key information about Zika prevention that they learned from television and the internet. Some of our participants could not avoid travel endemic areas and therefore, prevention strategies such as protection from mosquitos and barrier protection during sexual intercourse should also be emphasized.

Our findings are largely consistent with a quantitative study of Zika knowledge in NYC which found that among pregnant women in NYC (N=47), most heard of Zika from messaging in radio, TV, posters, and newspapers (71%), followed by internet and social media (35%) [12]. Although it does not breakdown these different educational platforms further, it does support our finding of TV as an important exposure to Zika information. It also shows that limiting Zika education to healthcare settings will lead to limited spread of information as only 13% reported receiving information from healthcare settings. The importance of social media was not observed in our study but should be further studied as a promising tool to disseminate emerging health information to young pregnant women who use social media technology in their daily lives.

Infections, like Zika, are emerging more frequently as is evidenced by the coronavirus 2019 (COVID-19) outbreak. Since the completion of the study, we have been grappling with the COVID-19 pandemic. The highly contagious infection with stay-at-home orders were enforced across the world created new access and communication barriers for patients. Unlike our experience with Zika, patients had limited access to healthcare providers in the early stages of the COVID-19 pandemic. The internet and social media became the primary source of information and disinformation about COVID-19. Leveraging social media and other technologies was deemed necessary if we are to provide accurate information to patients. One limitation to social media and the internet that have led to harm is what one author calls “infodemic” of misinformation and biased information that has limited the impact of our efforts to delivery medically accurate information to the population [12]. Another study evaluated COVID-19 pregnancy information on YouTube, and found high view rates but low quality and trustworthiness of the content [13]. Even in this environment, one report recommended prioritizing dissemination of information using a patient-centered framework with culturally and linguistically concordant messaging through social media, email, and phone [14].

While our objective was to understand how pregnant women learn about Zika, we also learned how to educate vulnerable populations about emerging infections. As one participant reminded us, knowledge is power, and empowering our community by improving its knowledge about health starts by analyzing where it has struggled in the past. The new Zika epidemic of 2016 and 2017 allowed for an evaluation of current systems of health education in the Bronx. It revealed a lack of knowledge, understanding, and prevention in the community, with medical interactions occurring after risk of exposure had occurred, instead of before [15]. When a new epidemic such as Zika occurs, it is essential to widen messaging and exposure to information, not only via mainstream sources such as TV, but also via social media, billboards, and community messaging.

Our research also identified barriers faced with Zika health education and identified new opportunities for education in the future. A summary of recommendations based on our findings can be found in Table 3. The dissemination of information about emerging infections is of critical importance to protect the health of vulnerable populations such as pregnant women in the Bronx. Our analysis and recommendations reveal that timely dissemination could occur through television and internet and that clinicians and healthcare providers are still critical in clarifying means of prevention and informing patients of the potential dangers these infections pose.

**Table 3:** Recommendations by pregnant women for community dissemination of health information in the context of emerging infections

Consider the full scope of individuals who are getting messages from TV and provide information for everyone	
Be sure that information is being disseminated in many languages so that multiple generations of family members can obtain and spread culturally appropriate information within their community and family	
Think about what information is available via a Google search and try to assure that it is from a trusted source. Consider providing links to trusted sources on TV and through healthcare resources.	
Consider greater use of social media and pregnancy apps to spread trusted reliable information about emerging infectious diseases. Consider the role of nationally prominent as well as locally trusted influences such as famous celebrities as well as family and friends.	
Further engage community resources frequently used by pregnant women such as WIC centers and schools in order to spread emerging health information. Investing and utilizing local infrastructure is crucial in settings such as the Bronx, where local messaging may empower community members to educate themselves and others.	

## Conclusion

This work was carried out during the Zika epidemic of 2016 -17, but it has renewed relevance because it can assist us in understanding ways to overcome the barriers to infection prevention that pregnant women experienced based on a lack of knowledge, understanding, and prevention in the community. Analysis of educational behaviors highlighted the wide reaching impact of TV and Google/Internet in creating awareness and easily accessible information with clinicians and healthcare providers delivering critical guidance and validation of this information. We learned from these behaviors and interactions that health dissemination and community guidance for vulnerable populations in emerging epidemics must utilize multiple sources beyond just the healthcare setting and including leveraging the internet and social media to deliver accurate information to the population at large.

## Supplementary Information

**Additional file 1: Appendix 1:** Supplemental TV Quotes

**Additional file 2: Appendix 2:** Supplemental Quotes about Internet / Google

**Additional file 3: Appendix 3.** Supplemental Healthcare Provider Quotes

**Additional file 4: Table S1.** Common Questions Used about Zika Education

## Data Availability

The datasets used and/or analyzed during the current study are available from the corresponding author on reasonable request.
