# Supplementary material for: Educational behaviors of pregnant women in the Bronx during Zika’s International emerging epidemic: “First mom … and then I’d Google. And then my doctor”
Source: BMC Pregnancy Childbirth. 2021 Oct 26;21:719. doi: 10.1186/s12884-021-04170-0 (PMC8547288; doi:10.1186/s12884-021-04170-0)
Supplement: Supplementary file 1 — Additional file 1: Appendix 1: Supplemental TV Quotes [file 12884_2021_4170_MOESM1_ESM.docx]

**Appendix 1:** Supplemental TV Quotes

**Supplemental** **Quotes about Zika Education via TV**

Main Themes:

- TV played the role of exposure in educating our participants and community about Zika.
- TV, especially news broadcasts and Spanish language channels, was a main source of information for our patients, their families, friends, and community, engaging all ages.
- TV was furthermore seen as a trusted source of information with great power to activate community and individual level concern and action about preventing Zika.
- Barriers to Zika education via TV included the limited information provided, information only provided during the height and hype of the epidemic, and dramatic presentations of Zika scaring pregnant women and causing denial to cope with the anxiety.

**Quotes about News**

“The 6:00 noticiero show. So, they’ll show you, you know, partners that had babies and they show you the pictures and they show you the actual [born] baby… And then they’ll – they’ll educate you and show you the bullet points, like don’t travel outside of New York if you’re pregnant and if you are, then these are the things that you should do...So – and so, that’s all in there like what to watch, how to watch, how to be careful before the pregnancy when we’re trying to get pregnant.” (Z4) (39 year old participant, partner traveled to Mexico)

“I heard it on television… There are Dominican channels.” (29 year old woman, traveled to Dominican Republic)

“On television. I remember it came out on the news…I think it was Telemundo 47.” (18 year old participant, traveled to Dominican Republic)

**Quotes about family watching TV.**

“Yes, for sure. My mother believes everything she hears on TV but has no other way to look it up. I’m like, “Ma, you have a phone…You can ask,” but she refuses to. It’s whatever is on TV is what – it’s the truth. ….. Right, correct. She’s older and she watches TV everyday.” (28 year old participant, husband traveled to Puerto Rico)

“I don't know [where my friends and family learn about Zika]. Maybe the TV?” (25 year old participant, traveled to Ecuador)

“I think my mom would know more about this than me. She watches [Telemundo] – she knows a lot about like diseases and, ‘You ought to be careful,’ and she knew, too, that we’re trying to get pregnant. And she was the number one person that kept reminding me about Zika…. she’s like, ‘Oh, I have to call my daughter. Oh, my god. This is going to – oh my god. I saw another. There was another case,’ and she would tell me where the case was and how old were they and like yeah.” (39 year old participant, partner traveled to Mexico)

**Quote about when people saw it on TV.**

“I think…. What should I say?   It’s always the same with any publicity campaign they do. People ignore it. They don’t think they need to waste their time looking at it. And they don’t trust it. They always think it’s all about money. But, later, when more news comes out on television, that’s when they want information.” (18 year old participant, traveled to Dominican Republic)

‘[I knew] all of this just from a flyer [seen at the clinic]. And then I started watching it on TV and I'm like, ‘I know about this. You see, I told, I told you about Zika.’ [Laughter] And everybody was like calling me, ‘What’s that that’s coming? Oh my God, everybody is telling about this.’ And, Yes, it’s great.” (31 year old participant, traveled to Honduras)

**Quote about only seeing when Pregnant and need to reach people before pregnancy**

“There, there on TV. It’s the Zika virus. [Laughter] When I was pregnant, so that I’ll run, and you know, up in the TV, I’ll watch the news of whatever clip it was on Zika. And I think like everything, and they’ll show like the map lighting up this country, this country, it’s like – it was like an auction, who has the most Zika. [Laughter] You know? So, it was – it was scary but everyone looked like – everyone was in the same page, you know? Everybody has the same general idea of what Zika is.” (31 year old participant, traveled to Honduras)

**Quotes about it rising and falling in the News**

“Maybe it has gone away.  I don’t know.  Because there was more talk before. They used to talk about this on the news, but now they are not talking about it.” (29 year old participant, traveled to Dominican Republic)

“They showed a lot of – when everything was starting, like that was maybe like last year or the year before that they show a lot of babies that were born with deformities, you know.” (25 year old participant, traveled to Ecuador)

“I think I remember one time, I think it was last year, they talked about it on television, on the news. They don’t talk much about it now.” (36 year old participant, traveled to Dominican Republic)

“Nobody is talking about this out on the streets.  I don’t see anybody talking about this out there.  I don’t see anything on television.  There’s nothing being said about this mosquito.” (29 year old participant, traveled to Dominican Republic)

**Quotes about not watching TV**

“So I don’t have time to be watching the news all day to find out new things that are going down.  Like I find out a little bit late about things that are happening.” (22 year old participant, traveled to Mexico)

“Honestly, I don’t really watch TV. I do Hulu and Netflix… on Fire Stick so I don’t really watch, you know news, like that. So – but a lot of Hispanics they do watch like, you know, Hispanic channel.” (25 year old participant, traveled to Ecuador)

**Quote about TV lacking information.**

“I didn’t realize that that was a uhm – a place that Zika was – had – has been infected. And it was the Caribbean Island so I know it was, you know, with some mosquitos but not upset with Zika ‘cause I hadn’t it heard on the news but there are no - there were cases in the US but never in Saint Thomas. So, but that’s why I'm here. It’s like that’s why I wanted to get tested just to make sure, you know. It’s for precaution purposes, so it’s going to be okay.” (30 year old participant, traveled to Saint Thomas)

“If you rate the disease by the effects, so that’s how it gets on the news, just killing children or making them so sick they’re losing weight, turning yellow. But if you treat all of that in time, you can prevent certain things.” (21 year old participant, traveled to Honduras)

“You only hear about it when it’s like, ‘Oh we have a case of Zika because someone travelled outside the US.’ And then they’re like this whole, big news story for months and then it goes away. And then you don’t hear about what, you know - how this affects their child uhm, you know, how has it affected the parent, like the livelihood of the mother, the – the uhm, the livelihood of the – of the – of the child, whether or not the child will survive.” (30 year old participant, traveled to Saint Thomas)

**Quotes about Denial and Fear**

“In the news they show the worst part of it – you know, only the birth” (25 year old participant, traveled to Ecuador)

“Sometimes they show things on the news that you think won’t happen to you, that you’re somehow safe.” (36 year old participant, traveled to Dominican Republic)
