# Supplementary material for: Educational behaviors of pregnant women in the Bronx during Zika’s International emerging epidemic: “First mom … and then I’d Google. And then my doctor”
Source: BMC Pregnancy Childbirth. 2021 Oct 26;21:719. doi: 10.1186/s12884-021-04170-0 (PMC8547288; doi:10.1186/s12884-021-04170-0)
Supplement: Supplementary file 2 — Additional file 2: Appendix 2: Supplemental Quotes about Internet / Google [file 12884_2021_4170_MOESM2_ESM.docx]

**Appendix 2:** Supplemental Quotes about Internet / Google

**Table 6: Supplemental Quotes about Zika Education via Internet / Google**

Main Themes

- The Internet and Google played a role of research database in educating our participants and community about Zika.
- The internet and Google was a main source of information for pregnant women to look up information and to share information with friends. Our participants used the internet / Google because of how quickly available the information could be found and the many topics they could research especially on their phone.
- Problems with relying on the internet and Google, included contradicting ideas and not knowing what sources to trust. Researching on the internet could be a scary and isolating experience lacking social support.

**Quote about Google being best Friend / main source of information**

“[I look up medical information] on the internet. Yeah, pretty much my best friend. That’s about it. The internet.” (21 year old participant, traveled to Honduras)

“Yeah. [I look up medical information on] Uhm, Google is my best friend. So, I Google everything.” (39 year old participant, partner traveled to Mexico)

“First the mom [who, found out via TV] and then I’d Google. And then my doctor ...Any questions that I have that I wanna ask the doctor…I’ll just ask to Google and I’ll try to learn more that way.” (39 year old participant, partner traveled to Mexico)”

**Quote about using Google because only source of info**

“[When I am on the internet, I] read Google. [I am not going to ask anyone] because I’m pretty sure nobody around me is gonna know just like – just like I didn’t.” (30 year old participant, traveled to Puerto Rico)

“No, [I am not going to ask anyone about Zika] because I think I get better information searching the Internet or asking a professional.” (29 year old participant, traveled to Dominican Republic)

**Quote about phone**

“[I get my health information] on my phone. [I use]Google. [Laughter] Honestly, because if you – I don’t know. People just don’t know but I look to find articles or whatever on what’s going on, to find out. That’s what I – that’s what I personally do. ‘Cause I’m always on my phone or on the go. Uh, like usually it’s like MSNBC. Like, uhm, CNN. I look for those. But on CNN, they talk about – they were talking about Ebola and everything. Usually, outbreaks and stuff like that, they’re the first ones.” (22 year old participant, traveled to Mexico)

“On the Internet. Yes, when I’m looking for something I use my cellphone to go to websites.” (29 year old participant, traveled to Dominican Republic)

**Quote About Speed**

“Someone that you confide in. Like the sort that you go to over time. I think that, yeah. You should go to that person [for medical information], but I feel like the media and technology is in the palm of your hands. So, I think it’s like it’s so easy to just – just type it and you just research it. You could do some research of it.” (39 year old participant, partner traveled to Mexico)

**Quote about using google to share images.**

“I had to go online and show him what could possibly happen- I had to show him pictures…of what could possibly happen”(22 year old participant, traveled to Mexico)

**Quote about appointments and using Google to search**

“Every time I go [to a doctor’s appointment], I know that if I want to learn more, I’ll have to look up the information [on Google]. That’s just how I am. That’s me.” (36 year old participant, traveled to Dominican Republic)

“I use Google. I just go to Google. I just Google Zika: ‘What is like Zika virus?’ and ‘How to prevent Zika?’ …or like any questions that I have that I wanna ask the doctor [later in my appointment]…I’ll just ask to Google and I’ll try to learn more that way.” (39 year old participant, partner traveled to Mexico)

**Quotes about Contradicting or un-reliable information on the Internet / Google**

“I feel when people start Googling things or doing internet research, it’s not reliable.” (28 year old participant, partner traveled to Puerto Rico)

“That’s a problem because you don’t know whether you can trust a site or not. Sometimes you read contradictory ideas.” (36 year old participant, traveled to Dominican Republic)

**Quotes about being Scared by information on the Internet / Google**

“I just didn’t trust going on the internet and then it makes me more paranoid so I’d rather just stay away.” (28 year old participant, partner traveled to Puerto Rico)

“I went to Google. I mean, uhm, it was pretty scary because it becomes graphic. They show pictures of the baby’s birth defect.” (25 year old participant, traveled to Ecuador)

**One Participant had much to say about being Scared by Information on the Internet:**

“Like I did a whole bunch of research on Google and that’s like scary because they were showing you pictures and you know, the small-shaped heads that babies get. And, you know, all these medical issues that a child could possibly get if you get bitten by Zika - like a mosquito that has Zika. So, it was very scary, it was very scary.

Very - you know, pessimistic about everything. And, you feel like if you have one bump, you already have cancer, you know? So, it’s very scary. So, all my research that I did was on Google and it just said, ‘You know, if I get this, you get infected. Zika’s in your blood, your baby comes out deformed, and you have all these medical issues, and what - your child has medical issues and you’re fine but not your baby.’ And you know, ‘Your baby won't talk or eat normally,’ or, you know, stuff like that. So, it’s very scary. It’s very scary.

I did like research, like I said, I was in Google. So, it’s like all negative. They give you no positive… [Laughter] …outcomes on Google. All those horrible things they showed me…It’s as if you’re going to die, or - you know what I mean?

Yeah, Google. Like I just – I just have to – I don’t know. I think that’s like my source of info. Like if I need something, I’ll go to Google and they’ll give something. And, I know that Google’s going to probably give me a ten [much more severe disease] and it’s like a five [less severe disease], but I know that’s going to give – it’s going to open my mind. It’s going to give me some type of information that I didn’t know before, or scare the crap out of me about something that I didn’t know and I'm going to prevent it. Or, you know, stuff like that. I'm actually grateful that I’ve – I’ve learned what I learned with my previous pregnancy, because I was so paranoid, but she came out perfect, you know, so yeah” (31 year old participant, traveled to Honduras)
