# Supplementary material for: Educational behaviors of pregnant women in the Bronx during Zika’s International emerging epidemic: “First mom … and then I’d Google. And then my doctor”
Source: BMC Pregnancy Childbirth. 2021 Oct 26;21:719. doi: 10.1186/s12884-021-04170-0 (PMC8547288; doi:10.1186/s12884-021-04170-0)
Supplement: Supplementary file 3 — Additional file 3: Appendix 3. Supplemental Healthcare Provider Quotes [file 12884_2021_4170_MOESM3_ESM.docx]

**Appendix 3:** Supplemental Healthcare Provider Quotes

**Table 5: Supplemental** **Quotes about Zika Education via Health Care Providers**

Main Themes

- Healthcare providers played a role of guidance in educating our participants and community about Zika.
- Although not immediately available, they were a trusted source of information, providing personal support, and helping to validate or dismiss information found online.
- Participants also advised healthcare providers to be more direct when educating about Zika and avoid relying solely on pamphlets that patients might not read.

**Quotes about Healthcare providers as trusted sources of information**

“I know that the information is available. I want to get it from a reliable source which would be a doctor… [the doctor gave] me a paper on Zika so I read that. I was comforted by that pamphlet that they gave me.” (28 year old participant, partner traveled to Puerto Rico)

“After they [ the doctors] talked to me a bit about it [Zika], I started searching on Google, and that made me worry a little more… but there’s nobody in my family, I don’t know of anyone who has had a problem [with Zika], so, I wasn’t so terribly worried. But, now that I am pregnant, and my doctor talked to me, it seems more important to me.” (36 year old participant, traveled to Dominican Republic)

“You could tell me anything, but if I’m looking – if there’s something direct that I can read and it tells me that this is what could happen, I’m going to follow that, and I’m going to follow [Laughter] those posters and what my doctor says.” (37 year old participant, traveled to Puerto Rico)

“[If I wanted to learn more about Zika] I’d get in touch with a medical center that could provide information.” (29 year old participant, traveled to Dominican Republic)

“Definitely, [Any medical question I have] I will ask her [my doctor]- in my next appointment… If I want to find out more, I would ask my doctor and I’ll ask when I go back to my next appointment… She [my doctor] never gives me a website. Pretty much everything that I ask her, she gives me answer for it.” (30 year old participant, traveled to Puerto Rico)

“I did but I feel – this is my theory, again. I feel when people start Googling things or doing internet research its not reliable – number one. Number two, they [my doctor] give me a paper on Zika so I read that. I was comforted by that pamphlet that they [my doctor] gave me. I just didn’t trust going on the internet and then it makes me more paranoid so I’d rather just stay away… She [My mom] was one of the people that say, ‘The government is trying to scare you.’ I’m like, ‘No, it’s my doctor. I’m not speaking to a government official.’ ” (28 year old participant, partner traveled to Puerto Rico)

“My gynecologist, every time I went, always [I saw information about Zika]… I had an appointment with him before I left, and I also saw signs and posters with information about the Zika virus, and this information is still there when I go to the gynecologist… So, I read more things. My gynecologist said, ‘Look, here’s an informational campaign about Zika’. I have an appointment with the pediatrician tomorrow, and every time I go, they ask me about it.” (18 year old participant, traveled to Dominican Republic)

**Quotes about using healthcare providers to validate research information**

“So, I did research and then I spoke to my doctor.” (39 year old participant, traveled to Mexico)

“I don’t know [if what I read online is a fact or rumor]. I’m just gonna read it and keep it in here. So when I speak to my doctor, I know.” (30 year old participant, traveled to Puerto Rico)

**Quotes about advice for Healthcare Providers on how to Educate about Zika**

“[The doctor says] ‘here’s some information. This is Zika.’ [But she should also say] ‘This is a picture of what would happen if you get infected by Zika.’ …I think they should be more adamant about letting us know…I think they [doctors] feel like maybe they [pregnant women] already knew because it’s been out there so much like on social media and stuff with, you know, TV and stuff like that, the news. That I think they feel like we already know.” (31 year old participant, traveled to Honduras)

“I suppose that if you have a doctor’s appointment, any doctor’s appointment, it doesn’t matter what sort of doctor’s appointment, the pediatrician or other doctor should give you information.  He/she should talk to you, not just hand you a piece of paper, because, as I said, you can give me a paper and I can have it, but I’m probably not going to read it. Because a lot of papers don’t tell you anything important.” (18 year old participant, traveled to Dominican Republic)

“I think every doctor - first, even it’s just a physical or for anything, any doctor should tell you, ‘If you’re trying to conceive, you know, be aware,’ you know. So I think – so like every appointment, any doctor you go to they should inform you about it.” (30 year old participant, traveled to Puerto Rico)
