# Supplementary material for: Educational behaviors of pregnant women in the Bronx during Zika’s International emerging epidemic: “First mom … and then I’d Google. And then my doctor”
Source: BMC Pregnancy Childbirth. 2021 Oct 26;21:719. doi: 10.1186/s12884-021-04170-0 (PMC8547288; doi:10.1186/s12884-021-04170-0)
Supplement: Supplementary file 4 — Additional file 4: Table S1. Common Questions Used about Zika Education [file 12884_2021_4170_MOESM4_ESM.docx]

Data / Supplemental Tables

**Table S1: Common Questions Used about Zika Education**

|  | **Questions** |
| --- | --- |
| 1 | Where did you learn that information about Zika? |
| 2 | Where do you recommend posting Zika Information? / how to educate others about Zika? |
| 3 | Where do you go to look up info about Zika? |
| 4 | Did you find that information useful? / do you trust the information? |
| 5 | What were barriers for you to access information to Zika? |
